# Challenges of Neoantigen Targeting in Lynch Syndrome and Constitutional Mismatch Repair Deficiency Syndrome

**DOI:** 10.3390/cancers13102345

**Published:** 2021-05-13

**Authors:** Asima Abidi, Mark A. J. Gorris, Evan Brennan, Marjolijn C. J. Jongmans, Dilys D. Weijers, Roland P. Kuiper, Richarda M. de Voer, Nicoline Hoogerbrugge, Gerty Schreibelt, I. Jolanda M. de Vries

**Affiliations:** 1 Department of Tumor Immunology, Radboud Institute for Molecular Life Sciences, Radboud University Medical Center, 6525 GA Nijmegen, The Netherlands; asima.abidi@radboudumc.nl (A.A.); Mark.Gorris@radboudumc.nl (M.A.J.G.); evan.brennan@student.ru.nl (E.B.); Gerty.Schreibelt@radboudumc.nl (G.S.); 2 Princess Máxima Center for Pediatric Oncology, 3584 CS Utrecht, The Netherlands; m.c.j.jongmans-3@umcutrecht.nl (M.C.J.J.); D.D.Weijers@prinsesmaximacentrum.nl (D.D.W.); r.kuiper@prinsesmaximacentrum.nl (R.P.K.); 3 Department of Genetics, University Medical Center Utrecht, 3584 CX Utrecht, The Netherlands; 4 Department of Human Genetics, Radboud University Medical Center, 6525 GA Nijmegen, The Netherlands; Richarda.deVoer@radboudumc.nl (R.M.d.V.); nicoline.hoogerbrugge@radboudumc.nl (N.H.); 5 Department of Medical Oncology, Radboud University Medical Center, 6525 GA Nijmegen, The Netherlands

**Keywords:** Lynch Syndrome, hereditary cancer, CMMRD, neoantigen, colorectal cancer, mismatch repair deficiency, targeted therapy

## Abstract

**Simple Summary:**

Lynch syndrome (LS) and constitutional mismatch repair deficiency (CMMRD) are hereditary disorders which significantly increase a person’s risk of developing a variety of cancers such as colorectal, endometrial, brain and, for CMMRD also, haematological malignancies. This increased cancer risk is due to inherited mutations in specific types of DNA repair genes, which hampers repair of mispaired or damaged bases during DNA replication. As a consequence, somatic mutations rapidly accumulate and typically include insertions and deletions (indels) in microsatellites that potentially can give rise to neoantigens. These neoantigens open up avenues for neoantigen-targeting immune therapies. Here, we aim to discuss the major obstacles that are encountered in developing such a therapy, including the heterogenous tumour profile of LS and CMMRD patients which challenge the selection of suitable neoantigens and potential resistance to immune checkpoint inhibitor therapy. In addition, we give a perspective on how to overcome the encountered obstacles.

**Abstract:**

Lynch syndrome (LS) and constitutional mismatch repair deficiency (CMMRD) are hereditary disorders characterised by a highly increased risk of cancer development. This is due to germline aberrations in the mismatch repair (MMR) genes, which results in a high mutational load in tumours of these patients, including insertions and deletions in genes bearing microsatellites. This generates microsatellite instability and cause reading frameshifts in coding regions that could lead to the generation of neoantigens and opens up avenues for neoantigen targeting immune therapies prophylactically and therapeutically. However, major obstacles need to be overcome, such as the heterogeneity in tumour formation within and between LS and CMMRD patients, which results in considerable variability in the genes targeted by mutations, hence challenging the choice of suitable neoantigens. The machine-learning methods such as NetMHC and MHCflurry that predict neoantigen- human leukocyte antigen (HLA) binding affinity provide little information on other aspects of neoantigen presentation. Immune escape mechanisms that allow MMR-deficient cells to evade surveillance combined with the resistance to immune checkpoint therapy make the neoantigen targeting regimen challenging. Studies to delineate shared neoantigen profiles across patient cohorts, precise HLA binding algorithms, additional therapies to counter immune evasion and evaluation of biomarkers that predict the response of these patients to immune checkpoint therapy are warranted.

## 1. Introduction

Lynch Syndrome (LS) is an autosomal dominantly inherited disorder resulting from monoallelic germline aberrations in genes that are involved in DNA mismatch repair (MMR) machinery [1]. The four MMR genes that are implicated in the disorder are *MLH1*, *MSH2*, *MSH6* and *PMS2* [2]. Patients with LS inherit a pathogenic germline variant in only one allele while the remaining wild type allele is somatically inactivated by point mutations, loss of heterozygosity or epigenetic silencing due to promoter hypermethylation [3,4]. In 1999, two reports described the phenotype within LS families including children who carried homozygous germline mutations in the *MLH1* gene. The children in both families displayed haematological malignancies in early childhood and clinical features that were previously known from neurofibromatosis type 1 [5,6]. Since then, individuals who inherit bi-allelic germline mutations in one of the MMR genes have been identified to suffer from constitutional mismatch repair deficiency (CMMRD). This rare syndrome is inherited recessively with homozygous or compound heterozygous germline mutations in the DNA MMR genes, most commonly *PMS2* and *MSH6* [7].

**Figure 1:**
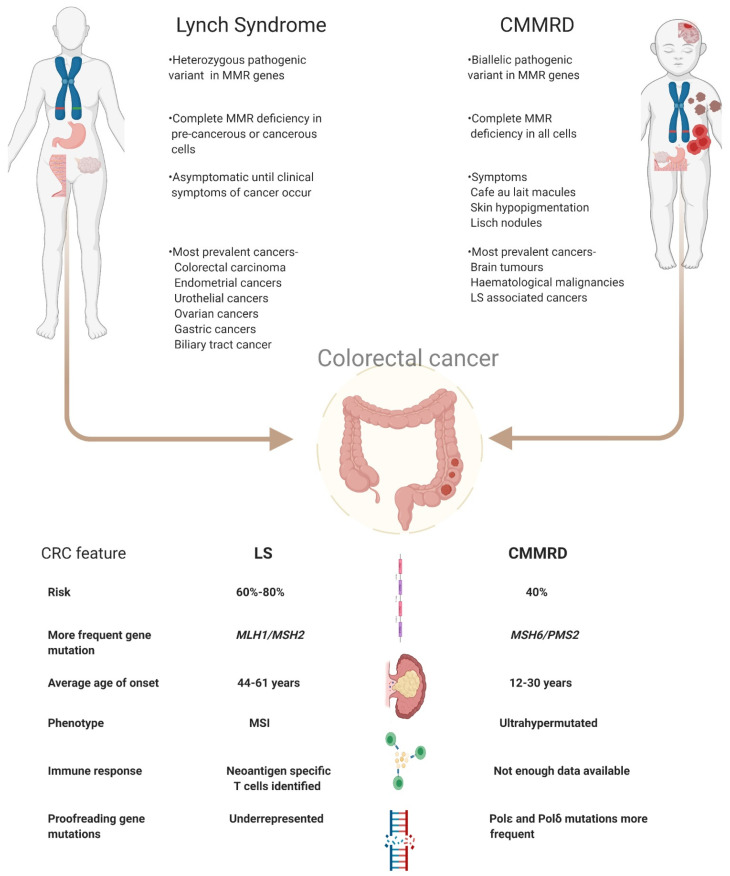
Schematic view on the differences between LS and CMMRD with a focus on colorectal cancer. Abbreviations used- LS: Lynch Syndrome, CMMRD: Constitutional mismatch repair deficiency syndrome, MMR: mismatch repair, CRC: colorectal cancer, MSS: microsatellite stable, MSI: microsatellite instable.

LS increases a person’s risk of colorectal cancer (CRC) by 40–80% and endometrial cancer by 15–60% [8]. Individuals with LS are also more prone to a variety of cancers among which are urothelial (0.4–20%), ovarian cancers (4–12%), gastric cancers (<10%), brain tumours and also cancers of the biliary tract [9,10,11,12]. Similarly, CMMRD patients have an increased risk of developing CRC in adolescence or young adulthood. In patients with CMMRD 50% develop malignant brain tumours while 40% develop cancers of the digestive tract [13]. The risk of developing haematological malignancies is as high as 30% [13]. In fact, the penetrance of cancers in CMMRD is one of the highest among childhood cancer syndromes, and it is extremely uncommon for a patient not to have developed cancer by the third decade [14]. The increased cancer risk in LS patients stems from the loss of the second functional MMR allele which results in accumulation of somatic mutations leading to carcinogenesis [15]. In contrast, tumorigenesis in CMMRD patients does not depend on second hit mutations since the biallelic loss of MMR functioning itself renders the cells unable to repair damaged DNA and hence lose genomic integrity [16]. Figure 1 summarises the key features of LS and CMMRD.

The accumulation of somatic mutations and genomic instability, especially in mutation prone regions, e.g., regions of repetitive nucleotide sequences, results in non-synonymous mutations. These mutations give rise to proteins with altered amino acid sequences called frameshift peptides (FSPs) that can give rise to neoantigens [17,18]. Neoantigens make an attractive target for immunotherapies since they have not been subjected to central and thymic tolerance and are solely expressed by tumour cells [19]. Tumours with high mutational burden such as those in LS and CMMRD patients are more likely to give rise to neoantigens and hence provide more opportunities for targeted therapies [18]. Despite the presence of technological facilities that help with the efficient identification of such neoantigens, therapies henceforth developed are still in nascent stages when compared to neoantigen targeting therapies in melanoma which have shown tumour regression in patients [20]. This demands further probe into the aspects that are impairing a successful neoantigen targeting regimen in LS and CMMRD. This review aims to assess the challenges that neoantigen targeting in LS and CMMRD is currently facing with a perspective on overcoming them.

## 2. Mismatch Repair Deficiency and Microsatellite Instability

DNA damage can occur endogenously through metabolic processes inside a cell and through exogenous processes like environmental agents. The repair pathways involved in the repair of damaged DNA are broadly classified as base excision repair, homologous recombination, non-homologous end joining, nucleotide excision repair and MMR [21,22]. The role of the DNA MMR system is to maintain genomic integrity through base pair and small insertion-deletion (indel) corrections that are erroneously generated during DNA replication [23]. The most important components of the DNA MMR system are the MutS and MutL complexes. In its functional state, MutSα, consisting of MSH2 and MSH6 proteins, recognises single base indels. Functional MutSβ, consisting of MSH2 and MSH3 proteins, recognises indels consisting of 2–8 nucleotides. MutLα, consisting of MLH1 and PMS2, or MutLβ, consisting of MLH1 and PMS1, binds together with the MutS complex as a heterodimer along with replicative factors to initiate repair of the mismatched nucleotides. Since MSH2 and MLH1 are the proteins shared by both components of their respective MutS and MutL complexes, a mutation in the respective genes will completely retard all MMR activity whereas a mutation in *PMS2* or *MSH6* genes will reduce MMR activity towards single nucleotide indels only [24]. Tumours arise from clonal expansion of cells that have inactivation of both alleles of a MMR gene that can either be somatic or of germline origin.

**Figure 2:**
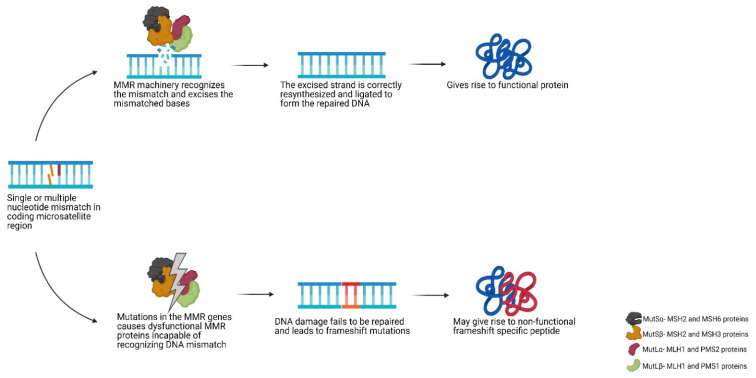
Schematic view of the DNA mismatch repair pathway. Abbreviations used- MMR: mismatch repair.

Indels commonly occur in regions of repetitive nucleotide sequences called microsatellites, where the template and the primer strands are prone to slippage (i.e., dissociation and re-annealing) during replication. Such mismatches are not repaired in MMR deficient cells, resulting in an incorrect number of repeat units between the template and newly synthesised strand. The microsatellite alterations can lead to a shift in the translational reading frame and therefore generation of FSPs [25]. This genetic alteration is termed microsatellite instability (MSI) and is a characteristic of LS-associated cancers. The process is briefly summarised in Figure 2. MSI is not specific for LS and CMMRD, approximately 15% of sporadic colorectal cancers also demonstrate MSI that most often originates by hypermethylation of the *MLH1* promotor and somatic bi-allelic inactivation of MMR genes [26,27,28]. While all microsatellites have an equal chance for mutations, differences in their mutation frequency can occur due the length of the repeat and the nature of the relevant nucleotide sequence.

## 3. Clinical Management of LS and CMMRD

Since LS patients are at risk of early onset CRC, regular colonoscopy surveillance starting from age 20–25, is essential to diagnose early lesions with the intent to prevent development of CRC. For LS patients, regular coloscopy is quite a burden and does not prevent the formation of new lesions, pointing to the need for other preventive measures [29]. There is evidence of reduced CRC risk in LS patients and sporadic MMR gene mutation carriers who took 600 mg/day aspirin for at least 2 years [30]. However, there are concerns regarding the risk of bleeding events in young patients [31]. Since a subset of chemotherapeutics rely on a functional MMR system to induce tumour damage, the efficacy of such drugs in MMR-deficient tumours such as in LS or CMMRD has been poor [32]. In fact worse prognosis was seen for stage II MSI high (MSI-H) colon cancer patients in a randomised trial administering adjuvant based 5-Flurouracil chemotherapy (5-FU) as compared to microsatellite stable (MSS) tumours, as a result of a lesser effectiveness of 5-FU in these MSI-H cancers [33]. The resistance of MMR deficient cells to drugs such as temozolomide, an alkylating agent used to treat glioblastoma multiforme (GBM), can lead to a greater risk of developing second primary tumours in CMMRD patients because of the accumulation of unrepaired mutations [7]. MMR-deficient cells are also more resistant to cisplatin treatment in comparison to MMR-proficient cells [34,35].

MSI-H cancers have a higher density of infiltrating lymphocytes compared to MSS cancers, which has been demonstrated to correlate with a better prognosis [36,37]. This observation highlights the potential of immunotherapy. In fact, LS-associated cancers have more pronounced local immune responses as compared to sporadic MSI-H cancers [38]. However, increased infiltration is counteracted by increased checkpoint protein expression which is an important mechanism by which the tumour microenvironment inhibits immune responses. By chronically expressing checkpoint receptors such as CTLA-4, PD-1, TIM-3, LAG-3 and more, T-cells become functionally exhausted and dysfunctional [39]. The upside of this checkpoint protein overexpression phenotype is the strong efficacy of immune checkpoint inhibitor therapies that have shown positive outcomes in MMR-deficient tumours across a range of malignancies [40]. This outcome has already led to FDA approval of the PD-1 antibody pembrolizumab for the treatment of refractory dMMR/MSI-H solid malignancies, and the PD-1 antibody nivolumab with or without the CTLA-4 antibody ipilimumab for the treatment of dMMR/MSI-H CRC after 5-FU treatment [41]. In a trial combining nivolumab and ipilimumab (NCT02060188), a disease control rate of 79% for >12 weeks was reported irrespective of clinical LS history [42]. Phase II studies have shown the safety and durable efficacy of nivolumab in patients with advanced MMR-deficient CRC (NCT01876511) where an immune-related progression free survival rate was seen for 78% of patients with MMR-deficient cancers as compared to the 11% of patients that had MMR-proficient cancers. However, it is important to note that while objective responses were seen in 100% of all non-LS-associated MMR-deficient cancers, only 27% (3 of 11 patients) of LS-associated cancers showed an objective response [40]. In a case study of a patient with LS-associated metastatic CRC, pembolizumab treatment reduced the metabolic activity of the cancerous lesions and improved symptoms [43]. Additionally, another case report from a LS patient with pancreatic adenocarcinoma and metastatic liver disease showed excellent clinical response with regards to liver lesion shrinkage after only one cycle of pembrolizumab treatment [44]. Nivolumab has also been reported to have significant efficacy at inducing an anti-tumour response and prolonging survival for two patients with CMMRD recurrent glioblastoma [45]. In another young child with CMMRD-associated GBM, nivolumab therapy showed a 60% reduction in tumour size and improved symptoms [46]. These groundbreaking results have amplified interest in the potential use of checkpoint inhibitors in combination with other therapeutics in the treatment of MMR-deficient cancers in LS and CMMRD.

## 4. Targeting Neoantigens in LS and CMMRD

Tumours that arise due to LS or CMMRD are highly mutated compared to their MMR-proficient countertypes. For instance, paediatric glioblastomas in CMMRD patients exhibit an ultra-high number of nonsynonymous mutations (≥250 mut/Mb) which contrasts the low frequency of nonsynonymous mutations (<1 mut/Mb) seen in the majority of glioblastomas [47,48].

The high tumour mutational burden (TMB) generated because of the MMR defect can result in neoantigen formation. These neoantigens are formed when indels result in a frameshift of the amino acid sequence in the C-terminal of the protein producing an FSP, that acts as a substrate for antigen processing and presentation via the major histocompatibility complex (MHC) class I and II molecules [49]. Once presented on the cell surface, they are referred to as neoantigens and could act as targets for tumour-infiltrating CD4^+^ helper T lymphocytes and CD8^+^ cytotoxic T lymphocytes (CTLs). Various clinical trials employing neoantigen based therapies for CRC are already ongoing [50].

Several studies have identified neoantigens in LS patients that are highly immunogenic, for example TGFβRII, CASP5, TAF1B, HT001 and OGT [51,52]. More importantly, CTLs specific to these neoantigens have also been detected in LS patients. CTLs directed against TGFβRII and CASP5 neoantigens are capable of lysing MSI-H colon carcinoma cells as shown in in-vitro assays [52,53,54]. Some of these mutations are found to be shared between LS and non-LS MSI-H CRCs hence calling attention to neoantigen-targeting therapies encompassing more patient groups [55]. Moreover CTLs specific for a wide range of neoantigens have been found to be induced already in patients that have not yet developed a cancerous lesion i.e., in healthy LS carriers [52]. This points towards the strong immune surveillance mechanisms in LS patients whereby the immune system recognises and possibly has the potential of eradicating MMR-deficient cells even before they develop into cancer. These observations strongly argue in favour of a promising efficacy of neoantigen-targeting therapy for therapeutic and preventive purposes in LS and CMMRD with added significance for prophylactic purposes to prevent admission of chemotherapy.

Strong lymphocyte infiltration in MMR deficient cancers makes it a prime target for checkpoint inhibitor therapy, some of which have been discussed above. In addition, it opens avenues for other immunotherapies such as dendritic cell (DC) vaccination. DC vaccination may lead to the specific enhancement of immune responses against neoantigens and hence lesser toxicity as opposed to general immune activation in response to checkpoint inhibition. This is already being investigated in a DC vaccination trial in LS mutation carriers, to assess the feasibility of DC vaccination loaded with frameshift derived neoantigens associated with MSI (NCT01885702). Preliminary data show that after DC vaccination, neoantigen-specific T-cells are detectable in blood and delayed type hypersensitivity (DTH) tests, without the induction of severe adverse events [56]. Similarly, in another Phase I/II trial (NCT01461148) in LS patients, a vaccine against the neoantigens HT001, AIM2 and TAF1B has been shown to be well tolerated with no severe adverse effects in any patient, and induced humoral and cellular responses in all patients [57]. Another recent case study investigated the use of a combination of autologous DCs producing IL-12, nivolumab (anti-PD1 receptor) and radiotherapy for the treatment of a CMMRD patient that showed a complete therapeutic response [58]. Another promising immunotherapeutic approach was demonstrated preclinically, with the adoptive transfer of CTLs with an engineered TCR-directed against a FSP of the *TGFβRII* gene. This particular *TGFβRII* frameshift mutation is reported in 90% of MSI-H CRC [59]. The adoptive transfer induced significant reduction in tumour load in a xenograft mouse model [60]. However, this approach has not been tested in humans yet.

As promising as these strategies are, there are still many obstacles that need to be overcome.

## 5. Challenges of Neoantigen Targeting in LS and CMMRD

**Figure 3:**
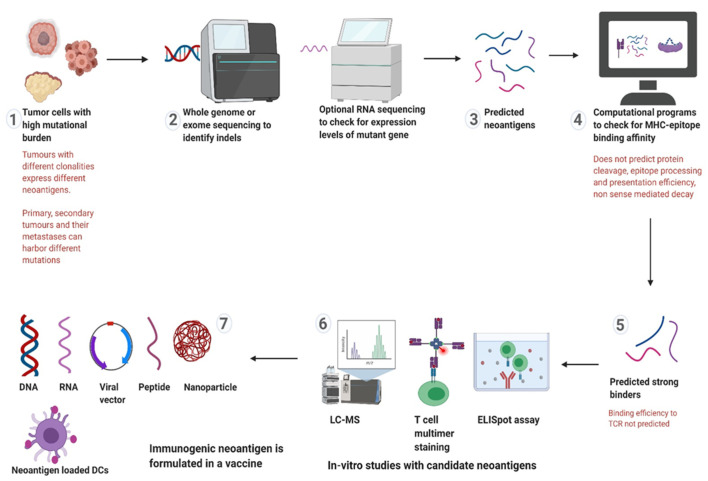
Schematic view of the different steps of developing a neoantigen targeting therapy and the challenges encountered in each step. 1–2 Whole exome sequencing from resected tumours or adenomas identifies indels; 3–4 The predicted neoantigens are assessed for HLA binding affinity through machine-learning methods; 5–6 Predicted strong binders are tested for immunogenicity in-vitro through biochemical and immunological assays; 7 Immunogenic neoantigen can be formulated in different forms before being administered in the patient. Abbreviations—TCR: T cell receptor, LC-MS: Liquid chromatography-Mass spectrometry.

The development of a neoantigen vaccine basically begins with the identification of suitable neoantigens through sequencing techniques and computational modelling. The predicted neoantigens are then synthesised chemically and tested for recognition by CTLs. The subsequent design and delivery of the vaccine is optimised and tested preclinically depending on the type of cancer it is targeting and several other factors [18,61]. The process is detailed in Figure 3. For a vaccine to mount the most potent anti-tumour response, it must induce a significant increase in the quantity of neoantigen-specific T cells that have a high affinity to this particular neoantigen. This is a complicated task and multiple challenges are encountered.

### 5.1. Tumour Heterogeneity

Heterogeneity of tumours can be classified into (1) interpatient heterogeneity which refers to the differences in the clinical manifestation of the same type of tumour in different patients (2) intertumoural heterogeneity which refers to the acquisition of a different set of mutations and other histological and clinical characteristics in metastatic lesions or second primary tumours compared to the primary tumour and (3) intratumoural heterogeneity which refers to the different phenotypes and genotypes of groups of cells that form the same tumour [62]. Since LS gives rise to tumours with complex carcinogenic pathways, all three heterogeneities have implications for neoantigen-targeting.

Interpatient heterogeneity in LS/CMMRD patients is associated with the MMR gene mutation seen in the patient. Several studies have come to a consensus in defining significant differences in cancer risks associated with mutation of MMR genes. While mutations in *MLH1* were mostly indicative of an increased risk of CRC, *MSH2* and *MSH6* mutations were seen to predispose to extracolonic malignancies such as endometrial and urothelial cancers in addition to CRC and *PMS2* to a lower life time cancer risk and more atypical malignancies [63,64]. The heterogeneity in tumour manifestation is also seen in CMMRD cases where *MLH1/MSH2* mutations predominantly give rise to, but are not limited to, highly aggressive haematological malignancies, while *MSH6* and *PMS2* mutations mostly give rise to brain tumours before the age of 10 years. Forty percent of the patients with homozygous *PMS2* mutations give rise to second primary malignancies, while only 22% of patients with homozygous *MLH1/MSH2* develop second primary malignancies. This is because patients with homozygous *MLH1/MSH2* develop more aggressive malignancies and have much lower chance of surviving the first malignancy as compared to *PMS2* mutation carrier patients [13]. The striking interindividual heterogeneity in LS-associated CRC also results from differences in molecular pathways involved [36]. It was initially described that MMR deficiency was a secondary event that enhanced tumorigenesis of pre-formed polyps resulting from *APC* mutations [15]. On the contrary, recent evidence from MMR-deficient crypt foci (MMR-DCF) has pointed to two more carcinogenic pathways which describe MMR deficiency as the initiator and not the accelerator of carcinogenesis. MMR-DCF can give rise to (1) cancers developing from intra-mucosal neoplastic lesions after accumulation of mutations in genes such as *APC*, *KRAS* or *RNF43*, and (2) immediately invasive cancers arising from non-polypus lesions through mutations in *CTNNB1* or *TP53* genes [65]. It is also of significance to note that while hypermethylation pattern and MSI tumours are enriched on the right side of the colon, not all tumours on the right side of the colon are hypermutated [66]. Moreover, although MMR deficiency itself is a common event for all carcinogenic pathways described in LS-CRC, patients have heterogenous risks of developing hypermutated polyps which highly express neoantigens or polyps with a low mutational burden and therefore a lower expression of neoantigens depending on the mutation of the MMR gene and the carcinogenic pathway involved [50,67]. In an interesting study among LS-CRC patients a contrast was seen that divided the patients in two groups where a majority of tumours in the first group had *MLH1* mutations and consequently greater MS slippages leading to higher TMB. In contrast, the majority of tumours in second group had *MSH2* mutations, lesser DNA damage and lower TMB resembling an MSS-sporadic CRC phenotype [68]. Hence predicting a neoantigen profile that is widely shared among most patients is more difficult. A study comparing MSI patterns between adult MMR deficient tumours and paediatric CMMRD patients, found that adult MMR deficient tumours exhibited highly mutated microsatellite loci hotspots while paediatric tumours from CMMRD patients did not. For instance, the mutation in microsatellite region of gene *ACVR2* is shared among 45% of adult MMR deficient tumours but only 11% of paediatric tumours from CMMRD patients [69].

Intertumour heterogeneity was observed in an LS-CRC patient in whom the first tumour was shown to be MSH2 positive but another more proximally located tumour was completely MSH2 deficient [70]. For CRC cases in general, a discordance has been shown between the mutations harboured in primary tumours and metastases. In a few CRC cases, the primary tumour has been shown to have a MSS phenotype while the corresponding liver metastasis were highly mutated with apparent MSI [71]. This certainly demands more studies into the mutational profile of metastases in LS-CRC patients.

Presence of intratumoural heterogeneity in LS-CRC has been highlighted by the fact that the resulting neoantigens have variable expression patterns such that not a single neoantigen is known to be present on all MSI cells [52]. Despite this overwhelming heterogeneity, neoantigens arising from mutations in *OGT*, *TGFBR2*, *CASP5*, *BAX*, *ASTE1*, *ACVR2*, *TAF1B*, *PTEN* are considered to be common as they have been shown to be present in >50% of MSI-H tumours [52,72]. A recent study concerning 10 LS and 5 CMMRD patients identified mutations in the coding regions of *RNASET2* and *TFDP1* genes shared among and between majority of LS and CMMRD patients in their small cohort. This paves the way for shared neoantigen identification and subsequent targeting [73].

Since heterogeneity works to the tumours’ advantage, an interesting suggestion in this regard is to increase a tumours homogeneity by selecting for a tumour clone that is resistant to initial treatment and further treating it with immunotherapy so that the immune system has a more homogenous target [74]. This also provides scope for an initial off-the-shelf immunotherapy targeting shared neoantigens. Moreover, it has been shown that DC tumour infiltration is significantly greater in MSI-H CRCs compared to MSS CRCs, this could play a role in epitope spreading by diversification of the immune response to other antigens presented by the tumour [75]. Recently, the role of circulating tumour DNA and circulating tumour cells as liquid biopsies is being explored as a non-invasive means of predicting metastasis and response to therapy for CRC. For instance, circulating tumour DNA can shed light on the heterogenous tumour landscape and molecular characterisation of the circulating tumour cells can help in the identification of tumour clones resistant to therapy [76]. This is a promising tool that must be investigated in LS and CMMRD patients as well.

However, choosing a suitable neoantigen in CMMRD patients is particularly difficult because, in contrast to LS patients in whom only (pre)malignant cells are completely MMR-deficient, in CMMRD patients MMR deficiency is present in all cells and therefore the neoantigens could be expressed in healthy cells of the patient as well. Vaccination must therefore be done with caution so as to not induce a major auto-immune response.

### 5.2. Neoantigen Selection

As indicated in step 1 of Figure 3, neoantigen identification starts with sequencing the tumour exome and optional RNA sequencing to identify the indels in microsatellite regions which are then analysed through computational modelling to predict the neoantigens that have the strongest binding affinity to the patients’ MHC molecules [77]. Among the various prediction models, NetMHC is the most commonly used software to predict the MHC:peptide binding affinity. This model relies on an extensive list of affinity measures described by the Immune Epitope Database and Analysis Resource (IEDB) and on the database for MHC ligands and peptide motifs (SYFPIETHI) [78]. The IEDB has curated information from over 700,000 experiments to reflect the binding of an epitope specific TCR or MHC molecule to an experimentally tested antigen and the SYFPEITHI contains a dense collection of various MHC class I and II molecules and peptide motifs [79]. While a sizeable number of sequencing studies has been conducted for LS related and sporadic MSI-H cancers providing data for such simulations, data of CMMRD related cancers is still sparse.

In spite of the availability of extensive data on MHC molecule structures, the prediction models fail to account for the various other steps of epitope processing such as the efficiency of the MHC ligand processing and the abundance of the precursor protein giving rise to the neoantigen itself. Additionally, some transcripts can be sensitive to non-sense mediated decay in which case they are not expressed by the mutant cells [80]. In addition, the protein cleavage and the efficiency of the transporter associated with antigen presentation (TAP) play a major role in peptide-MHC binding, which is difficult to predict. Moreover, the neoantigen prediction models shed little light on cross-reactivity with self-peptides [81]. Some neoantigen cancer vaccine trials have shown that more polyfunctional CD4^+^ T cells than CD8^+^ T cells are induced in melanoma and glioblastoma patients despite the neoantigens being predicted to bind strongly to human leukocyte antigen I (HLA-I) molecules [82]. This has been attributed to the promiscuous binding properties of HLA-II proteins among other factors. While this points towards the ambiguity of MHC binding prediction algorithms, this effect is not undesirable because CD4^+^ T cells have been shown to aid in tumour clearance and similar effects must be investigated in LS/CMMRD vaccination trials as well.

Since most prediction models mentioned above rely on HLA-binding strength of predicted neoantigens, the neoantigens with lower affinity to HLA complex but having high immunogenicity are often overlooked [81]. It is important to realise that neoantigens that have high recognisable potential might be unable to induce effective T cell responses because of being insufficiently present in the tumour [83]. There is also evidence for the presence of subdominant and cryptic neoantigens that are not capable of inducing an immune response naturally but can be activated through vaccination [84]. This could be an opportunity that is currently underutilised in searching for shared neoantigens in high mutational burden tumours found in LS and CMMRD.

In a study involving members of two unrelated families with LS, a neoantigen prediction algorithm called OncoPeptVAC was used to predict the immunogenicity of in silico derived peptides resulting from a common germline mutation in the MLH1 gene, observed in LS-associated cancer unaffected and affected members of both families [85]. This prediction algorithm also predicts the binding affinity of the neoantigen to the TCR and was able to predict that neoantigens resulting from the germline MLH1 mutation, would be non-immunogenic. It predicted that weaker binding of the neoantigen to the TCR outweighs the strong HLA binding on the antigen presenting cell, thereby rendering it non-immunogenic. This is preferable because neoantigens arising from germline mutations can be present in all cells and vaccination against these can trigger an auto-immune response. Moreover, using the same algorithm, neoantigens predicted from somatic mutations of the genes *MSH6*, *PIGO* and *AXIN2* observed in the LS-CRC tumour of one of the affected family members, was shown to induce interferon gamma (IFNγ) releasing T cell responses.

Another neoantigen prediction pipeline to analyse the distribution of frameshift mutations and their corresponding epitopes in MSI-H endometrial, colorectal, and stomach cancers revealed five FSPs of *SLC35F5*, *SEC31A*, *TTK*, *SETD1B* and *RNF43* genes that were shared among these cancer types, however no respective immunogenicity was tested [86]. Albeit, with the same pipeline, a total of nine FSPs for MSI-H endometrial cancers were discovered which had strong HLA-binding affinity and were also highly immunogenic. This predictability of immunogenic neoantigens was achieved by including longer peptides which encode multiple epitopes and by pooling the neoantigen-compatible HLA types resulting in a diverse poly-allelic HLA ligandome. However the presence of a high or low load of these shared polyepitope frameshift mutations was not found to be significantly associated with survival benefit of the respective patients [86]. Recently, a novel neoantigen-prediction tool called the REgression based FRAMEshift quantification algorithm (ReFrame) was developed that utilises a linear series of mathematical equations to account for stutter artifacts or the undesirable frameshift products that might result during slippage in the PCR amplification step [87]. Identification of indels arising from MSI was made more specific by this tool because it was coupled with a novel immunological scoring method. This method identifies immunologically relevant neoantigens by analysing the strength of binding to MHC and the prevalence of the corresponding MHC allele in a specific population. Since many different neoantigen prediction algorithms are now being developed, it is also important for the information to be shared among researchers to improve the features, sensitivity and accuracy of these algorithms. The Synapse platform developed by Sage Bionetworks is one such platform, which allows researchers to organise their data and codes and collaborate with other researchers.

It is also suggested to include hydrophobic and aromatic amino acid residues and differences in non-anchor residues to enhance immunogenicity [88]. Further biochemical assays can be used to determine peptide-HLA stability.

### 5.3. Vaccine Formulation

There are several different methods of formulating and delivering the neoantigen vaccine to a patient which include DNA-, RNA-, protein- or peptide-based delivery methods [86]. As mentioned above, for MMR deficient CRC patients, a phase I/II clinical trial with a peptide vaccine directed against AIM2, HT001 and TAF1B neoantigens employing the adjuvant Montanide ISA-51VG showed no severe toxicity in any of the patients while cellular and humoral immunogenic responses were seen in all 16 patients included in the study [57]. However, the authors do not comment on the efficacy of the vaccine in terms of tumour-specific cytotoxicity. This study suggests that protein/peptide-based vaccines require an adjuvant to induce immunogenicity though it is important to note that the partial uptake of only the peptide and not the adjuvant by the antigen presenting cells can be responsible for inducing tolerance instead of immunity [89]. Additionally, dendritic cells ex-vivo loaded with neoantigens as mentioned above are being explored in healthy LS and LS-CRC patients without serious adverse effects in most patients [56]. The response of peptide based vaccines can also be improved by taking longer peptides that code for multiple epitopes. RNA vaccines additionally activate TLR3, TLR7 and TLR8 responses mimicking adjuvant-induced inflammatory effects and are easier to produce than peptide vaccines but they are subject to easy degradation by RNAses and have reduced uptake by antigen presenting cells [90]. Encapsulation of synthetic DNA and RNA in nanoparticle formulation has been shown to prevent degradation, improve its bioavailability and facilitate the targeted and controlled delivery to antigen presenting cells [91,92].

Vaccine formulation consists of a challenging preclinical stage of development in which neoantigens are tested for their pharmacokinetic properties such as absorption, distribution, metabolism, excretion and clearance [93]. While small molecules can enter cells easily through diffusion, synthetic peptides undergo a complex set of processes that include peptide cleavage and presentation before finally being able to bind to their specific receptors on the cell [94]. This further complicates the process of conducting in-vitro or in-vivo pharmacological and immunological assays to track the pharmacokinetic properties of neoantigens. Perhaps this is the reason why, the testing for peptide toxicity in animal models is more often skipped and safety assessments are directly performed in phase I clinical trials in humans. Use of animal models for neoantigen targeting therapies is also particularly unfeasible because self-peptides and neoantigens may be significantly different between species. During the selection of neoantigens, it is prioritised to select for neoantigens that are shared among more patients. However, batch manufacturing for even a small set of patients requires GMP facilities. This process is still not very cost effective.

### 5.4. Immune Evasion and Immunosuppression

The potency of neoantigen targeting therapies in LS and CMMRD is hampered by the immune evasion mechanisms exerted by the tumour. If cells can evade eradication, equilibrium follows for a certain period of time [67]. Immune escape then eventually occurs which allows the remaining cells to evade detection by the immune system and undergo unrestrictive proliferation. Moreover, the downregulation of transcripts of highly immunogenic neoantigens to evade recognition is also seen [85]. Another overriding immune escape mechanism is the evidence for mutations in a microsatellite region of β2-microglobulin (*B2M*) gene which forms an essential component of the (neo)antigen presentation machinery of the MHC class I protein with the mutation leading to total loss of HLA expression observed in nearly 30% of MSI-H CRC patients [95,96,97]. Apart from *B2M* itself, mutations in the gene *NLRC5*, which is a direct transactivator of *B2M*, *TAP1* and *HLA A/B/C/D/E/F/G*, have been reported in MSI-H CRCs. This leads to the a disabled antigen presentation machinery, enabling immune escape, hence favouring expansion of tumour cells [98]. Additionally, frameshift mutations in *RFX5* observed in 40% of MSI-H CRCs and promoter methylation of *CTIIA* genes restrict IFNγ-inducible HLA class II antigen presentation [99,100]. While the B2M mutations predominate LS-CRC, inactivation of TAP1 or TAP2 proteins are more common in sporadic MSI-H CRC. This is important because it has an effect on the expression levels of additional HLA classes and also on NK cell activity [101].

Another interesting correlation between B2M mutation and immunosuppression is seen in the infiltration of Foxp3^+^ regulatory T cells (Tregs). In a study of LS-CRC patients it was shown that B2M wild type tumours had a higher frequency of Tregs and hence increased immunosuppression as compared to the B2M mutant tumours [102]. It was hypothesized that this may provide a survival benefit for the B2M mutant tumours in an environment where in spite of low infiltration of Tregs, the active immune system fails to induce a response by not recognising the tumour cells [102]. Moreover, in another study concerning an LS-CRC patient, a high density of immunosuppressive myeloid-derived suppressor cells (MDSCs) was seen. The authors hypothesised that MDSCs were the reason for the reduced activation of CD8^+^ T cells in the patient [85]. In this study, the authors also linked the increased infiltration of Tregs with a negative prognosis. This warrants further study into targeting MDSCs and Tregs in LS-CRC patients to overcome immunosuppression. Furthermore, it has been reported in a patient with MMR-deficient colon adenocarcinoma that NK cell activity has been affected by the increased frequency of M2 macrophages which interfere with NK degranulation, thereby hampering the response and shedding light on the immunosuppressive tumour microenvironment in MMR-deficient patients [41]. It is also to be noted that DC maturation is impaired in MSI-H CRCs that have high Treg infiltration which impedes neoantigen presentation in these patients [75], although vaccination with mature DCs, that are loaded with immunogenic neoantigens could potentially be a successful regimen.

**Figure 4:**
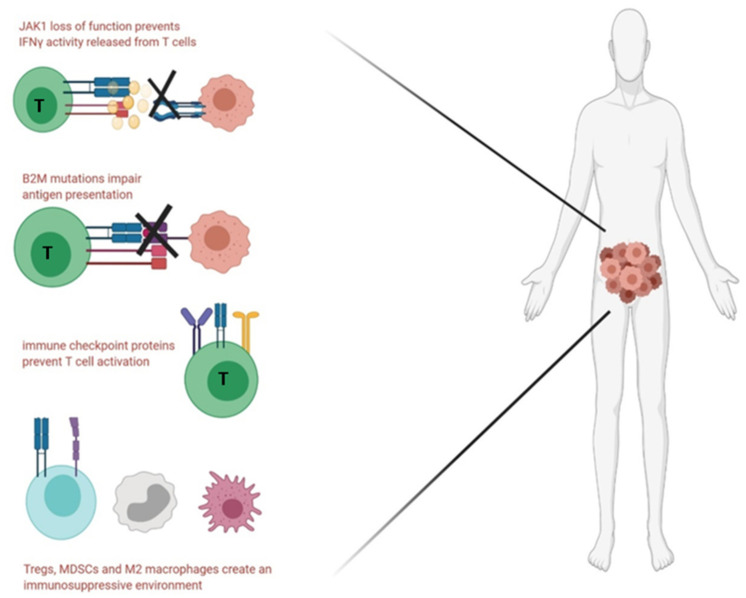
Brief overview of the immune escape mechanisms observed in MSI high CRC. Abbreviations—JAK-Janus Kinase, IFN: interferon, Treg: Regulatory T cells, MDSC: myeloid-derived suppressor cell.

A dominant channel to escape immune surveillance is the presence of immune checkpoint blockade molecules that can cause exhaustion of CD8^+^ T cells. These then fail to proliferate in response to antigen and they lack critical anticancer effector functions such as cytotoxicity and IFNγ secretion [39]. Interestingly, it has also been shown that B2M mutant MSI-H CRCs have a higher frequency of PD-1 positive T cells as compared to B2M wild type MSI-H CRCs, thereby further hampering the immune response [103]. While the promise of checkpoint inhibitor therapy in this regard has been discussed before, it is important to address the cases where the MMR-deficient CRC patients showed resistance or showed disease progression despite pembrolizumab treatment [104]. The sequencing of metastases from patients who developed progressive disease revealed mutations in B2M, though such mutations were not observed in the primary tumour. The lack of suitable biomarkers for predictability of the efficacy of immune checkpoint therapy is a major setback since 12% to 40% of all MMR deficient metastatic CRC patients show resistance, including acquired resistance in patients that were sensitive to the therapy initially [105]. Resistance to anti-PD-1 therapy in MSI-H CRC patients was attributed to homozygous loss of function mutations in IFNγ receptor Janus Kinase 1 (*JAK1*) [106]. It is hypothesised that the loss of JAK1 makes the tumour cells resistant to IFNγ secretion from cytotoxic T cells, thereby the dampening the anti-proliferative effect of IFNγ, as depicted in Figure 4.

An LS patient with metachronous urothelial and colon cancer being treated with anti-PD-1 therapy presented differential response to the therapy. Where no response was observed in the colonic lesion, pseudoprogression (increased tumour size during imaging due to infiltration of lymphocytes and macrophages which has positive connotations for immunotherapy) was seen for the ureteral lesion [107]. In the study concerning LS-CRC patients where two distinct patient groups were identified, the group of patients with high TMB and resembling sporadic MSI-H cancer type had lower expression of checkpoint genes such as *PD-1*, *PD-L1* and *CTLA-4* in the tumour as compared to the reference mucosa. It is hypothesised that the tumours in this subgroup have already undergone immune escape and have markedly lower infiltration of T cells thereby questioning the efficacy of immune checkpoint inhibition therapy in this subgroup [36,68]. Activation of the Wnt/β-catenin pathway in metastatic melanoma has been shown to reduce T cell infiltration and hence reduce IFNγ secretion as well as conferring resistance to immune checkpoint therapy, though similar studies in LS and CMMRD are lacking [108]. There is a scarcity of studies done to assess exclusive escape mechanisms or its implications in CMMRD patients and given the complexity of these suppressive processes, the need cannot be more emphasised.

A study demonstrated the effect of inhibition of cell cycle proteins cyclin dependent kinase 4 and 6 (CDK4 and CDK6) in upregulation of B2M and other proteins of MHC class I machinery. In addition it was found to increase infiltration of tumour-infiltrating T cells and decrease Tregs, the effects of which were enhanced by immune checkpoint therapy [109]. This is an encouraging proposal to look out for and test to enhance the efficacy of neoantigen targeting therapies in LS and CMMRD.

## 6. Conclusions

LS and CMMRD-related cancers provide a unique challenge to current treatment strategies with more potential for neoantigen-targeting therapies on account of high TMB. This is appealing for preventive purposes, since LS and CMMRD patients have a very high risk of developing cancer and LS associated tumours express shared neoantigens. These shared neoantigens have been shown to give rise to specific CTLs which are also present in healthy LS carriers [52]. However, it must be considered that preventive neoantigen vaccination could lead to selection of tumours that do not express these neoantigens anymore. In addition, there are major considerations for CMMRD patients for whom the predicted neoantigens could be present in all cells because all cells are MMR deficient. This calls for the identification of tumour-specific neoantigens which are not present in other MMR-deficient cells, to avoid the risk of significant toxicity. The overwhelming tumour heterogeneity in LS and CMMRD patients originating from MMR defect and giving rise to complex tumourigenesis pathways and diverse neoantigen repertoires, does not only make the choice of vaccine neoantigen more complicated but it also makes it tougher to predict response of the different tumours within the patient and across patient populations. The use of more accurate neoantigen immunogenicity prediction algorithms and neoantigen profiling across patients is required, since larger studies regarding neoantigen formation and immune responses in CMMRD patients is particularly lacking. It is then most suitable to combine a neoantigen targeting therapy to induce a potent T cell response with immune checkpoint therapy to overcome the immunosuppressive environment. Potential biomarkers in LS and CMMRD patients that can predict the response to neoantigen and immune checkpoint therapy are necessary since immune-evasion mechanisms in these cancers are complicated.

## Data Availability

Not applicable.
